# Outcome of Reoperation for Local Recurrence Following En Bloc Resection for Bone Giant Cell Tumor of the Extremity

**DOI:** 10.3390/curroncol29090503

**Published:** 2022-09-05

**Authors:** Shinji Tsukamoto, Andreas F. Mavrogenis, Suraj Hindiskere, Kanya Honoki, Akira Kido, Hiromasa Fujii, Tomoya Masunaga, Yasuhito Tanaka, Pramod S. Chinder, Davide Maria Donati, Costantino Errani

**Affiliations:** 1 Department of Orthopaedic Surgery, Nara Medical University, 840, Shijo-cho, Kashihara 634-8521, Nara, Japan; 2 First Department of Orthopaedics, School of Medicine, National and Kapodistrian University of Athens, 41 Ventouri Street, Holargos, 15562 Athens, Greece; 3 Department of Musculoskeletal Oncology, HCG Hospital, No. 8, P. Kalingarao Road, Sampangiramnagar, Bangalore 560027, Karnataka, India; 4 Department of Rehabilitation Medicine, Nara Medical University, 840, Shijo-cho, Kashihara 634-8521, Nara, Japan; 5 Department of Orthopaedic Oncology, IRCCS Istituto Ortopedico Rizzoli, Via Pupilli 1, 40136 Bologna, Italy

**Keywords:** giant cell tumor of bone, en bloc resection, denosumab, local recurrence, MSTS score

## Abstract

En bloc resection is typically performed to treat giant cell tumors of bone (GCTB), particularly when curettage can be challenging owing to extensive bone cortex destruction with soft tissue extension. Few reports have addressed the clinical outcomes after reoperation for local recurrence in patients with GCTB who underwent en bloc resection. In this multicenter retrospective study, we investigated local recurrence, distant metastasis, malignant transformation, mortality, and limb function in patients treated for local recurrence following en bloc resection for GCTB. Among 205 patients who underwent en bloc resection for GCTB of the extremities between 1980 and 2021, we included 29 with local recurrence. En bloc resection was performed for large tumors with soft tissue extension, pathological fractures with joint invasion, complex fractures, and dispensable bones, such as the proximal fibula and distal ulna. Local re-recurrence, distant metastasis, malignant transformation, and mortality rates were 41.4% (12/29), 34.5% (10/29), 6.9% (2/29), and 6.9% (2/29), respectively. The median Musculoskeletal Tumor Society score was 26 (interquartile range, 23–28). The median follow-up period after surgery for local recurrence was 70.1 months (interquartile range, 40.5–123.8 months). Local recurrence following en bloc resection for GCTB could indicate an aggressive GCTB, necessitating careful follow-up.

## 1. Introduction

A giant cell tumor of bone (GCTB) is an intermediate-grade primary bone tumor. Approximately 5% of all primary bone tumors are GCTB [1]; moreover, GCTBs commonly occur in the distal femur, proximal tibia, and distal radius [1]. GCTB have a high local recurrence rate (median, 20%) [2], 1–9% of GCTBs develop distant metastases [3,4,5,6,7,8], and approximately 2.4% of GCTBs develop malignant transformation (secondary malignant GCTB) [9,10]. The prognosis of malignant GCTB remains poor, with a reported mortality rate of 42–70% [11,12,13,14]. Curettage is the mainstay of treatment for preserving good limb function; however, it is associated with a relatively high local recurrence rate (median, 20%) [2]. En bloc resection (resection of a large bulky tumor virtually without dissection) should be considered in cases of extensive cortical destruction with extensive soft tissue involvement [15,16]. GCTB often extends close to the joints, necessitating resection of the joints and reconstruction with prostheses or allografts in extremities other than the proximal fibula and distal ulna [15,16]. En bloc resection and reconstruction with a prosthesis or allograft can reduce local recurrence rates (2–13%) compared to curettage; however, the postoperative function is poor, leading to more frequent complications, such as loosening of the prosthesis, fracture of the allograft, and joint subluxation [16,17,18]. Because tourniquets cannot be used for GCTB in the proximal femur or proximal humerus, the amount of bleeding is greater than that at other extremity sites. Hence, preoperative embolization is required [16,17].

Few reports have examined clinical outcomes after reoperation for local recurrence in patients with GCTB following en bloc resection [19,20,21,22,23]. Therefore, we performed a three-center retrospective study to investigate the rates of local recurrence, distant metastasis, malignant transformation, mortality, and limb function after reoperation for local recurrence of GCTB in the extremities following en bloc resection.

## 2. Materials and Methods

**Figure 1:**
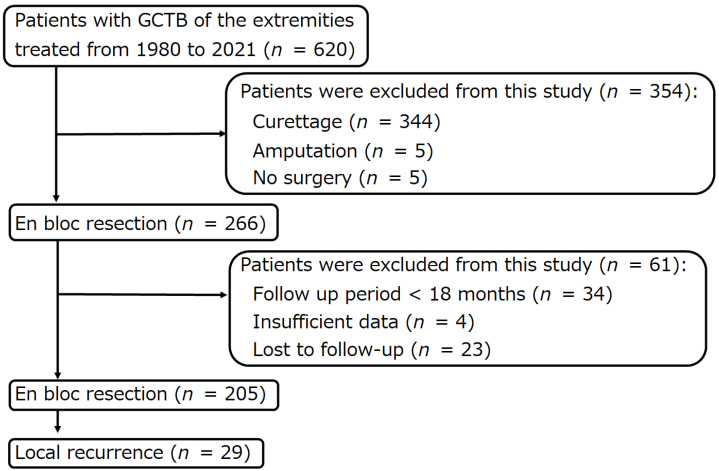
Flow diagram of patients with giant cell tumors of bones of the extremities, who were treated at three institutions between 1980 and 2021.

Among 620 patients with histologically diagnosed GCTB of the extremities treated at the authors’ institutions between January 1980 and December 2021, 29 patients with local recurrence after en bloc resection were retrospectively analyzed (Figure 1). The following data were collected from the patient’s medical records: age, sex, tumor site, Campanacci stage at presentation [24], lung metastasis at presentation, pathological fracture at presentation, denosumab administration before the first en bloc resection and before surgery for local recurrence, previous surgery at another hospital, time from the first en bloc resection to local recurrence, site of local recurrence (bone or soft tissue), surgical procedure for local recurrence, local re-recurrence, time from surgery for local recurrence to local re-recurrence, distant metastasis, malignant transformation, tumor death, postoperative follow-up period, oncological outcome (whether or not the tumor was observed at the final follow-up, whether or not the patient died of the tumor), Musculoskeletal Tumor Society (MSTS) score [25], surgery-related complications, and denosumab-related complications (Table 1 and Table 2).

Initial en bloc resection was performed for large tumors with soft tissue extension, joint involvement, complex fractures, or dispensable bones, such as the proximal fibula or distal ulna [26]. Overall, 4 patients underwent no reconstruction, 13 underwent allograft reconstruction, 8 underwent prosthetic reconstruction, 2 underwent free vascularized fibula graft reconstruction, 1 underwent arthrodesis by translocating the ipsilateral ulna as a vascularized graft [27], and 1 underwent reconstruction of the quadriceps tendon with mesh (Table 1). Local recurrence occurred in the bone and soft tissue of 11 (37.9%) and 18 (62.1%) patients, respectively. Curettage for local recurrence was performed in 3 of 29 patients (10.3%) with moderate cortical thinning and well-maintained bone structure [28,29]. Curettage was performed through a large cortical bone window using a sharp curette, and all visible tumors were removed [28,29]. Next, curettage was performed via the cavity using a high-speed bar, followed by washing with saline to remove the entire tumor [28,29]. Phenol was applied to the cavity border using a cotton-tipped applicator and neutralized with alcohol. Subsequently, the tumor cavity was filled with polymethyl methacrylate (PMMA) bone cement (Table 1). En bloc resection for local recurrence was performed in 25 of the 29 patients (86.2%), of whom 19 did not require any reconstruction, 4 underwent reconstruction with a prosthesis, and 2 underwent reconstruction with an allograft. Amputation was performed in one of the 29 patients (3.4%) with massive local recurrence surrounding the neurovasculature of the popliteal fossa (Table 1).

Denosumab was used to downstage the tumor by promoting the shrinkage of extraosseous lesions, tumor hardening, and osteosynthesis of pathological fractures (Table 1). Prior to the initial en bloc resection, two patients received denosumab (120 mg) subcutaneously, once weekly 1 one month, followed by once monthly for a total of 2–9 doses, depending on the clinical benefit, surgical plan, and clinical trial protocol (6 months). No denosumab was administered postoperatively. The patient received oral calcium (500 mg/day) and vitamin D (≥400 IU/day) supplements to prevent hypocalcemia. In addition, two patients received 1 or 29 doses of denosumab before surgery for local recurrence. En bloc resection was performed after the administration of denosumab (Table 1).

Patients underwent follow-up examinations (radiography of the tumor area and computed tomography of the chest) every 4 months for the first 2 years, every 6 months for the next 3 years, and annually thereafter. Local recurrence, lung metastasis, malignant transformation, and treatment-related complications were also recorded.

### Statistical Analysis

Local recurrence-free survival was defined as the period between surgery for local recurrence and local recurrence or last follow-up. Distant metastasis-free survival was defined as the interval between surgery for local recurrence and distant metastasis or the last follow-up. Malignant transformation-free survival was defined as the interval between surgery for local recurrence and the diagnosis of malignant transformation or the last follow-up. Disease-specific survival was defined as the interval between surgery for local recurrence and death from the disease or last follow-up. Local recurrence-free survival, distant metastasis-free survival, malignant transformation-free survival, and disease-specific survival were evaluated using Kaplan–Meier survival analysis, and survival curves were compared using a log-rank test. The data were analyzed using the JMP 14 software (SAS Institute Inc., Cary, NC, USA).

## 3. Results

**Figure 2:**
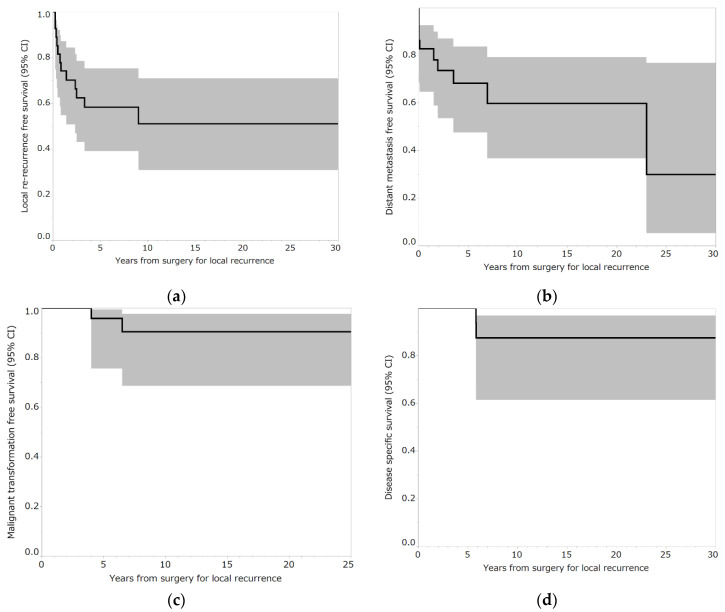
(**a**) Local re-recurrence-free survival rates of patients who underwent reoperation for local recurrence following en bloc resection for bone giant cell tumor of the extremities. Shading around the curves represents 95% confidence intervals (CI). (**b**) Distant metastasis-free survival rates of patients who underwent reoperation for local recurrence following en bloc resection for bone giant cell tumor of the extremities. Shading around the curves represents 95% CI. (**c**) Malignant transformation-free survival rates of patients who underwent reoperation for local recurrence following en bloc resection for bone giant cell tumor of the extremities. Shading around the curves represents 95% CI. (**d**) Disease-specific survival rates of patients who underwent reoperation for local recurrence following en bloc resection for bone giant cell tumor of the extremities. Shading around the curves represents 95% CI.

Local recurrence occurred in 12 out of 29 patients (41.4%). In the three patients who underwent curettage, local re-recurrence occurred in two patients (66.7%), whereas in the 26 patients who underwent en bloc resection, local recurrence occurred in 10 patients (38.5%). Five of the 11 patients (45.5%) who had local recurrence in the bone experienced local re-recurrence, whereas 7 out of 18 patients (38.9%) who had local recurrence in the soft tissue experienced local re-recurrence. The median time from surgery for local recurrence to local re-recurrence was 7.5 months (interquartile range [IQR], 4–25.3) (Table 2). The five-year local re-recurrence-free survival after surgery for local recurrence was 58.2% (95% confidence interval (CI): 39.0–75.2) (Figure 2a). Curettage for local re-recurrence was performed in two patients. En bloc resection for local re-recurrence was performed in 10 patients. None of the patients had a third local recurrence.

The distant metastasis rate was 34.5% (10 out of 29 patients). Two patients had lung metastases at presentation and two patients exhibited lung metastases at the time of surgery for local recurrence. Five patients had lung metastases and one had iliac metastasis after surgery for local recurrence. The median time from surgery for local recurrence to distant metastasis was 9.5 months (IQR, 0–52.3 months) (Table 2). The five-year distant metastasis-free survival after surgery for recurrence was 68.3% (95% CI: 47.5–83.7) (Figure 2b). Six patients underwent surgery for lung metastases. One patient with iliac metastases underwent curettage (Case 27) (Table 3 and Table 4). Two patients were followed-up for lung metastases. One patient experienced malignant transformation and died of the tumor (case 26) (Table 3 and Table 4).

The malignant transformation rate was 6.9% (2 out of 29 patients). The median time from surgery for local recurrence to malignant transformation was 13.5 months (IQR, 3–24 months) (Table 2). The five-year malignant transformation-free survival rate after surgery for local recurrence was 92.4% (95% CI: 74.1–98.1) (Figure 2c). (Table 3 and Table 4, respectively). One patient underwent external hemipelvectomy but experienced local recurrence and died due to the tumor (Case 5) (Table 3 and Table 4). The other patient underwent amputation and chemotherapy (cisplatin, ifosfamide, doxorubicin, gemcitabine, and docetaxel) but died after developing lung metastases (Case 26) (Table 3 and Table 4). The mortality rate was 6.9% (2 out of 29 patients). The median time from surgery for local recurrence to tumor death was 70 months (IQR: 70–70 months) (Table 2). Five-year disease-specific survival after surgery for local recurrence was 100% (Figure 2d).

The median MSTS score was 26 (IQR: 23–28) (Table 2). Surgical complications included two cases of infection requiring debridement and antibiotics (Cases 5 and 19) (Table 3 and Table 4) and one case of wrist arthropathy requiring arthrodesis (Case 6) (Table 3 and Table 4). One patient presented with an allograft fracture requiring allograft replacement (Case 3) (Table 3 and Table 4). No denosumab-related complications were noted (Table 2). The median follow-up period after the initial en bloc resection was 97 months (IQR, 66.4–141.5 months). The median follow-up period after surgery for local recurrence was 70.1 months (IQR, 40.5–123.8 months) (Table 2).

There were no significant correlations between local recurrence-free survival and variables, such as sex, age, tumor site, Campanacci stage at presentation, lung metastasis at presentation, pathological fracture at presentation, denosumab administration before the first en bloc resection, previous surgery, time from the first en bloc resection to local recurrence, location of local recurrence (bone or soft tissue), surgical method for local recurrence, denosumab administration before surgery for local recurrence, distant metastasis, or malignant transformation (Table 5).

## 4. Discussion

**Figure 3:**
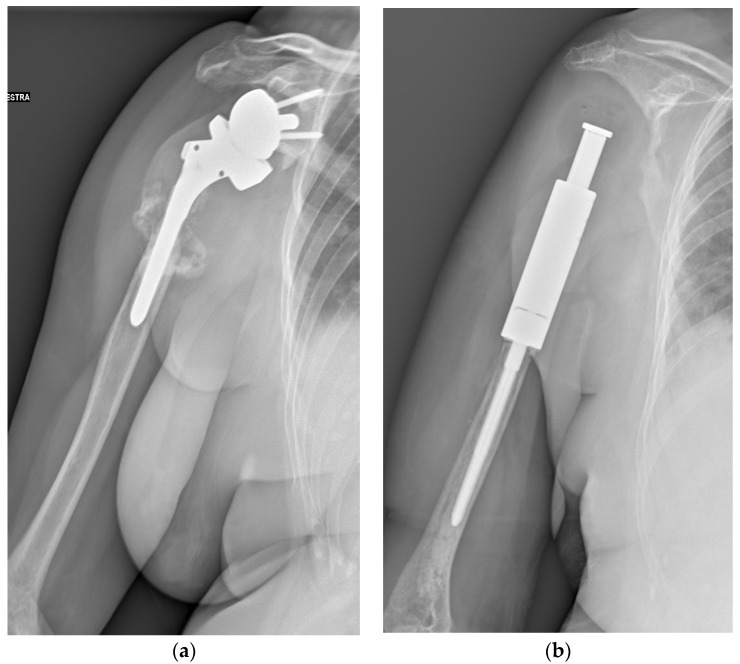

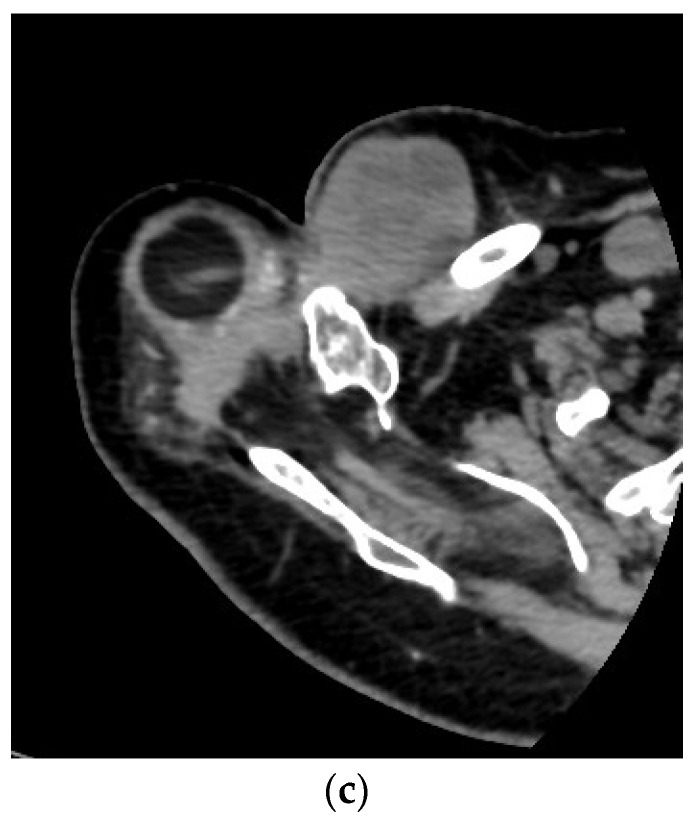
A 71-year-old female patient underwent en bloc resection and reconstruction with a prosthesis for a bone giant cell tumor of the proximal humerus with a pathological fracture at presentation. Six months postoperatively, she experienced local recurrence and was treated with denosumab for 2 years and 5 months (**a**). She then underwent tumor resection and revision of prosthesis (**b**). One year after the revision, she experienced local re-recurrence and underwent tumor resection. One month later, a third local recurrence and lung metastasis were detected, and the biopsy revealed malignant transformation (**c**). She died of the disease 6 years and 4 months after the first surgery (Case 26, Table 3 and Table 4).

Currently, there are few reports regarding the outcomes of reoperation for local recurrence after en bloc resection [19,20,21,22,23]. In this study, en bloc resection was performed for tumors with large extraosseous lesions, pathologic fractures with joint involvement, complex fractures, or dispensable bones, such as the proximal fibula and distal ulna. The local recurrence rate (41.4%), distant metastasis rate (34.5%), malignant transformation rate (6.9%), and mortality rate (6.9%) after reoperation for local recurrence after en bloc resection were higher than those reported in previous studies (local recurrence rate after initial surgery (median), 20% [2]; distant metastasis rate, 1–9% [3,4,5,6,7,8]; malignant transformation rate (median), 2.4% [9,10] and mortality rate, 1–1.7%) [11,12,13,14]. Therefore, recurrent GCTB after en bloc resection seems to exhibit markedly aggressive behavior and warrants careful follow-up after reoperation (Figure 3).

The local recurrence rate following en bloc resection in patients with GCTB is approximately 2–13% [19,20,21,22,23]. Prosser et al. reported that resection was performed in two patients with GCTB with local recurrence after en bloc resection, and local recurrence was observed in one patient (50%) [19]. Klenke et al. reported that 18 patients with GCTB that had locally relapsed after en bloc resection underwent resection, and one (6%) patient experienced local re-recurrence [20]. Niu et al. reported that a patient who experienced local recurrence after en bloc resection had an aggressive course [22]. The patient experienced multiple soft tissue recurrences after en bloc resection of GCTB of the distal ulna. He was treated with denosumab for 6 months and then underwent resection of multiple lesions but experienced multiple extensive soft tissue recurrences shortly after surgery and amputation [22]. A pathological examination revealed no malignant transformation. After the discontinuation of denosumab, multiple lung metastases showed significant progression [22]. Zhang et al. reported that a patient with GCTB of the proximal humerus experienced local recurrence in the soft tissue 18 months after receiving six doses of preoperative denosumab and en bloc resection. The patient remained disease-free for 9 months after undergoing en bloc resection for local soft tissue recurrence [23].

Higher local recurrence rates have been reported in the distal radius, proximal femur, hands, and feet [15,30,31]. The higher recurrence rate in the distal radius can be attributed to the relatively fragile bone quality of the distal radius, with the proximity of the distal radius to the carpal bones and ulna making adequate curettage more challenging [32,33]. The high recurrence rate in the proximal femur can be explained by insufficient curettage owing to the risk of osteonecrosis and fractures [15]. Furthermore, the high recurrence rate in the hand and foot could be attributed to difficulties in opening a large window, resulting in insufficient curettage, which makes it impossible to secure a sufficient field of view [30]. Herein, we detected no significant correlation between tumor site and local recurrence-free survival in patients with local recurrence after en bloc resection. Previous studies have reported that 9–30% of patients with GCTB may exhibit pathological fractures at presentation [31,34,35,36,37,38]. Patients with pathological fractures are often considered to have a more aggressive disease; however, a recent meta-analysis reported that pathological fractures have no significant effect on the local recurrence of GCTB [39]. In the present study, no significant correlation was observed between the presence of pathological fractures and local recurrence-free survival in patients with local recurrence after en bloc resection.

Furthermore, we found no association between the time from initial en bloc resection to local recurrence and local recurrence-free survival. Takeuchi et al. reported the prognoses of 94 patients with recurrence after initial curettage and those of 16 patients with recurrence after initial en bloc resection for GCTB of the extremities (110 patients). Of these, 25 patients experienced a second local relapse, and 6 patients had a third local relapse. The time from the first surgery to the first recurrence in the repeated recurrence group (two and three recurrences) was significantly shorter than that in the single recurrence group (mean 14.1 vs. 28.3 months, respectively; *p* = 0.016) [40]. Therefore, if the time between the first surgery and local recurrence is less than 24 months, the patient should be followed up carefully, given the high local re-recurrence rate after reoperation.

In GCTB, neoplastic stromal cells are rich in receptor activation of nuclear factor-kappa β (RANK) ligands, which induce receptor activation in RANK-positive osteoclast-like giant cells and their precursors [41,42,43,44,45,46,47]. Denosumab is a fully human monoclonal antibody that inhibits the RANK ligand (RANKL). It suppresses RANK-RANKL interaction and prevents bone destruction caused by giant cell tumors [45,48,49]. Denosumab was approved by the Food and Drug Administration in 2013, following reports of its observed efficacy and safety, although no other drugs have been approved for use in GCTB [50]. Denosumab has also been reported to exert a downstaging effect in less invasive surgery [51]. Currently, denosumab is indicated for the treatment of unresectable GCTB with significant functional impairment after resection [50]. In the case of GCTB with large soft tissue components in close proximity to neurovascular structures, the bony margins formed after denosumab administration can help reduce potential damage to neurovascular structures when the tumor and neurovasculature are dissected [52,53]. Denosumab may also facilitate intraoperative manipulation, prevent inadvertent tumor contamination, and decrease recurrence rates [52,53]. However, it has been reported that preoperative denosumab administration does not reduce the local recurrence rate after en bloc resection [53,54,55]. In this study, no significant correlation was observed between preoperative denosumab administration and local recurrence-free survival in patients with local recurrence after en bloc resection.

The outcome of lung metastases from GCTB varies from spontaneous regression to uncontrolled growth, with eventual death [56]. Mortality rates reported for metastatic GCTB range from 0 to 25% [5,6,7,57,58,59,60,61,62]. Lung metastases were significantly more common in patients with local recurrence [56]. In this study, no significant correlation was observed between the occurrence of lung metastases and local re-recurrence-free survival in patients with local recurrence after en bloc resection. Malignant GCTB is divided into primary and secondary types [9,13]. Primary malignant GCTB is characterized by the simultaneous presence of sarcoma and GCTB at initial diagnosis. Secondary malignant GCTB develops at sites of GCTB previously treated with surgery or radiotherapy (malignant transformation) [13]. Although malignant transformation was found to be significantly more common in patients with local recurrence [10], there was no significant correlation between the occurrence of malignant transformation and local recurrence-free survival in patients with local recurrence after en bloc resection in the present study.

This study had several limitations. First, indication bias for each treatment was associated with the retrospective study design. The small number of patients precluded multivariate analysis, and we could not adjust for confounding factors. Second, there is a possibility of type 2 errors due to the small number of cases. An increase in the number of patients could result in the emergence of factors with significant differences. In the future, a multivariate analysis with a large sample size is necessary. Third, the presence of the H3F3A mutation could only be confirmed in 4 out of 29 patients. Two of the four patients experienced malignant transformation, and no H3F3A mutations were observed in the malignant lesions. Therefore, the H3F3A mutation was not confirmed in the remaining 25 cases. These patients were diagnosed before evaluation. However, these patients were diagnosed by an experienced pathologist specializing in bone tumors.

## 5. Conclusions

The rates of local recurrence, distant metastasis, malignant transformation, and death after reoperation for local recurrence following en bloc resection are high. Therefore, GCTB that develop local recurrence despite en bloc resection are markedly aggressive and require careful follow-up.

## Figures and Tables

**Table 1:** Patients’ characteristics.

Variable ( *n* = 29)	No. (%) of Patients
Median age (years)	34 (IQR, 26.5–52.6)
Sex	
Male	13 (44.8)
Female	16 (55.2)
Site	
Distal radius	12 (41.4)
Proximal femur	2 (6.9)
Hand and foot	3 (10.3)
Distal femur	4 (13.8)
Proximal tibia	3 (10.3)
Proximal humerus	1 (3.4)
Others	4 (13.8)
Campanacci classification at presentation	
Stage II	4 (13.8)
Stage III	25 (86.2)
Lung metastases at presentation	
No	27 (93.1)
Yes	2 (6.9)
Pathological fracture at presentation	
No	24 (82.8)
Yes	5 (17.2)
Denosumab use at initial en bloc resection	
No	27 (93.1)
Yes	2 (6.9)
Previous surgery	
No	21 (72.4)
Yes	8 (27.6)
Median period from initial en bloc resection to local recurrence (months)	16 (IQR, 7.8–28.4)
Site of local recurrence	
Bone	11 (37.9)
Soft tissue	18 (62.1)
Surgery for local recurrence	
Curettage	3 (10.3)
Resection	25 (86.2)
Amputation	1 (3.4)
Denosumab administration before surgery for local recurrence	
No	27 (93.1)
Yes	2 (6.9)

IQR, interquartile range.

**Table 2:** Patients’ outcomes.

Variable ( *n* = 29)	No. (%) of Patients
Local re-recurrence	
No	17 (58.6)
Yes	12 (41.4)
Median period from surgery for local recurrence to local re-recurrence (months)	7.5 (IQR, 4–25.3)
Distant metastases	
No	19 (65.5)
Yes	10 (34.5)
Median period from surgery for local recurrence to distant metastasis (months)	9.5 (IQR, 0–52.3)
Malignant transformation	
No	27 (93.1)
Yes	2 (6.9)
Median period from surgery for local recurrence to malignant transformation (months)	13.5 (IQR, 3–24)
Median follow-up period from initial en bloc resection (months)	97 (IQR, 66.4–141.5)
Median follow-Up period from surgery for local recurrence (months)	70.1 (IQR, 40.5–123.8)
Oncological outcome	
NED (Local recurrence)	18 (62.1)
NED (Metastasis)	4 (13.8)
AWD (Metastasis)	5 (17.2)
DOD	2 (6.9)
Median period from surgery for local recurrence to DOD (months)	70 (IQR, 70–70)
Median MSTS score	26 (IQR, 23–28)
Surgery-related complication	
Infection	2 (6.9)
Wrist arthropathy	1 (3.4)
Fracture	1 (3.4)

NED, no evidence of disease; AWD, alive with disease; DOD, death from disease; MSTS, Musculoskeletal Tumor Society; IQR, interquartile range. NED (local recurrence) indicates no tumor after treatment for local recurrence. NED (metastasis) indicates no tumor after treatment for distant metastases. Patients who were tumor-free after treatment for both local recurrence and distant metastasis were classified as having NED (metastasis). AWD (metastasis) refers to alive with distant metastasis.

**Table 3:** Details of 29 patients with GCTB of the extremities who experienced local recurrence after en bloc resection.

Case	Sex	Age (Years)	Site	Campanacci Stage	Lung Metastasis at Presentation	Pathological Fracture at Presentation	Dose of Preoperative Denosumab	Reconstruction after Initial En Bloc Resection	Complication	Time to Local Recurrence (Months)	Site of Local Recurrence	Surgery for Local Recurrence	Dose of Denosumab before Reoperation
1	M	59	Metacarpal	Stage 3	No	No	0	Allograft	No	123	Soft tissue	En bloc resection	0
2	M	23	Distal radius	Stage 3	No	No	0	Allograft	No	16	Bone	Curettage	0
3	F	26	Distal radius	Stage 3	Yes	No	0	Allograft	Allograft fracture	6	Bone	Curettage	0
4	M	53	Proximal tibia	Stage 3	No	No	0	No reconstruction	No	23	Soft tissue	En bloc resection	0
5	M	63	Proximal femur	Stage 3	No	Yes	0	Prosthesis	Infection	27	Bone	En bloc resection and prosthesis	0
6	F	24	Distal radius	Stage 3	No	No	0	Allograft	Wrist arthropathy	11	Bone	En bloc resection and allograft	0
7	F	42	Distal femur	Stage 3	No	No	0	Prosthesis	No	21	Soft tissue	En bloc resection	0
8	F	54	Hand phalanges	Stage 2	No	No	0	Allograft	No	3	Bone	Curettage	0
9	F	51	Distal radius	Stage 3	Yes	Yes	0	Allograft	No	8	Soft tissue	En bloc resection	0
10	M	63	Proximal femur	Stage 2	No	Yes	0	Prosthesis	No	12	Bone	En bloc resection and prosthesis	0
11	M	52	Distal radius	Stage 2	No	No	0	Allograft	No	9	Soft tissue	En bloc resection	0
12	M	54	Distal radius	Stage 3	No	No	0	Allograft	No	23	Soft tissue	En bloc resection	0
13	F	23	Distal radius	Stage 3	No	No	0	Allograft	No	8	Soft tissue	En bloc resection	0
14	M	41	Distal ulna	Stage 3	No	No	0	No reconstruction	No	7	Bone	En bloc resection	0
15	M	38	Distal radius	Stage 3	No	No	0	Allograft	No	13	Soft tissue	En bloc resection	0
16	F	29	Proximal tibia	Stage 2	No	No	0	Prosthesis	No	16	Soft tissue	En bloc resection	0
17	F	28	Metacarpal	Stage 3	No	No	0	Allograft	No	30	Soft tissue	En bloc resection	0
18	M	22	Distal radius	Stage 3	No	No	0	Allograft	No	32	Bone	En bloc resection and allograft	0
19	F	50	Patella	Stage 3	No	No	0	Reconstruction of quadriceps tendon with mesh	Infection	36	Soft tissue	En bloc resection	0
20	F	30	Distal femur	Stage 3	No	No	0	Prosthesis	No	46	Soft tissue	En bloc resection	0
21	F	24	Proximal fibula	Stage 3	No	No	0	No reconstruction	No	52	Soft tissue	En bloc resection	0
22	M	40	Distal radius	Stage 3	No	No	0	Allograft	No	9	Soft tissue	En bloc resection	0
23	M	20	Proximal tibia	Stage 3	No	No	0	Prosthesis	No	16	Soft tissue	En bloc resection	0
24	F	29	Distal femur	Stage 3	No	No	0	Prosthesis	No	24	Bone	En bloc resection and prosthesis	0
25	F	31	Proximal fibula	Stage 3	No	No	0	No reconstruction	No	6	Soft tissue	En bloc resection	0
26	F	71	Proximal humerus	Stage 3	No	Yes	9	Prosthesis	No	6	Bone	En bloc resection and prosthesis	29
27	M	27	Distal radius	Stage 3	No	No	0	Vascularized fibular graft	No	18	Soft tissue	En bloc resection	0
28	F	28	Distal femur	Stage 3	No	Yes	0	Vascularized fibular graft	No	83	Bone	Amputation above knee	0
29	F	34	Distal radius	Stage 3	No	No	2	Translocation of the ipsilateral ulna	No	6	Soft tissue	En bloc resection	1

GCTB, giant cell tumor of bone; NA, not applicable.

**Table 4:** Details of 29 patients with GCTB of the extremities who experienced local recurrence after en bloc resection.

Case	Local Re-Recurrence	Interval between Surgery for Local Recurrence and Local Re-Recurrence (Months)	Surgery for Local Re-Recurrence	MSTS Score	Malignant Transformation	Interval between Surgery for Local Recurrence and Malignant Transformation (Months)	Distant Metastasis	Treatment for Distant Metastasis	Interval between Surgery for Local Recurrence and Distant Metastasis (Months)	Follow-Up Period from Initial En Bloc Resection (Months)	Follow-Up Period from Surgery for Local Recurrence (Months)	Status
1	No	NA	NA	20	No	NA	No	NA	NA	124	1	NED (RL)
2	Yes	17	En bloc resection	24	No	NA	No	NA	NA	83	67	NED (RL)
3	No	NA	NA	28	No	NA	Lung	Metastasectomy	0	42	36	AWD (M)
4	No	NA	NA	20	No	NA	No	NA	NA	70	48	NED (RL)
5	Yes	28	En bloc resection	17	Yes	24	No	NA	NA	97	70	DOD
6	Yes	30	En bloc resection	24	No	NA	Lung	Metastasectomy (twice)	0	139	128	NED (M)
7	No	NA	NA	29	No	NA	No	NA	NA	63	42	NED (RL)
8	Yes	3	Curettage	Unknown	No	NA	Lung	Observation	18	42	39	AWD (M)
9	Yes	3	En bloc resection	Unknown	No	NA	Lung	Observation	0	134	126	AWD (M)
10	No	NA	NA	25	No	NA	Lung	Metastasectomy	23	97	86	AWD (M)
11	Yes	10	Curettage	27	No	NA	No	NA	NA	47	38	NED (RL)
12	No	NA	NA	28	No	NA	No	NA	NA	81	58	NED (RL)
13	Yes	9	En bloc resection	24	No	NA	No	NA	NA	121	113	NED (RL)
14	No	NA	NA	30	No	NA	No	NA	NA	169	162	NED (RL)
15	Yes	5	En bloc resection	24	No	NA	No	NA	NA	77	64	NED (RL)
16	No	NA	NA	23	No	NA	No	NA	NA	167	151	NED (RL)
17	Yes	6	En bloc resection	23	No	NA	Lung	Metastasectomy (twice)	1	118	88	NED (M)
18	No	NA	NA	28	No	NA	Lung	Metastasectomy	83	223	191	NED (M)
19	No	NA	NA	28	No	NA	No	NA	NA	48	12	NED (RL)
20	No	NA	NA	28	No	NA	No	NA	NA	168	122	NED (RL)
21	Yes	4	En bloc resection	30	No	NA	Lung	Metastasectomy	0	73	20	AWD (M)
22	No	NA	NA	22	No	NA	No	NA	NA	90	81	NED (RL)
23	No	NA	NA	17	No	NA	No	NA	NA	18	3	NED (RL)
24	No	NA	NA	30	No	NA	No	NA	NA	144	120	NED (RL)
25	Yes	4	En bloc resection	28	No	NA	No	NA	NA	114	108	NED (RL)
26	Yes	40	En bloc resection	Unknown	Yes	3	Lung	Chemotherapy	42	76	70	DOD
27	No	NA	NA	Unknown	No	NA	Ilium	Curettage	276	327	309	NED (M)
28	No	NA	NA	Unknown	No	NA	No	NA	NA	446	363	NED (RL)
29	No	NA	NA	28	No	NA	No	NA	NA	51	45	NED (RL)

GCTB, giant cell tumor of bone; MSTS, Musculoskeletal Tumor Society; NA, not applicable; NED, no evidence of disease; M, metastasis; RL, local recurrence; AWD, alive with disease; DOD, death from disease.

**Table 5:** Univariate analysis for local re-recurrence-free survival in patients who experienced local recurrence after en bloc resection for GCTB of the extremities.

Variable	No. of Patients (*n* = 29)	5-Year Local Re-Recurrence-Free Survival (95% CI) (%)	*p*-Value
Sex			0.4647
Male	13	63.6 (33.9–85.7)	
Female	16	54.1 (29.8–76.7)	
Age (years)			0.9640
<30	12	54.5 (26.8–79.7)	
≥30	17	61.4 (36.2–81.6)	
Site			0.1566
Distal radius/Proximal femur/Foot and hand	4	100	
Others	25	52.8 (32.9–71.7)	
Campanacci classification			0.6924
Stage I, II	4	50.0 (12.3–87.7)	
Stage III	25	59.4 (38.5–77.5)	
Lung metastases at presentation			0.5086
No	27	59.1 (39.2–76.5)	
Yes	2	50.0 (5.9–94.1)	
Pathological fracture at presentation			0.5357
No	24	63.0 (41.5–80.4)	
Yes	5	40.0 (10.0–80.0)	
Denosumab administration before initial en bloc resection			0.9573
No	27	59.3 (39.4–76.6)	
Yes	2	50.0 (5.9–94.1)	
Previous surgery			0.3484
No	21	53.0 (31.3–73.7)	
Yes	8	71.4 (32.7–92.8)	
Period from initial en bloc resection to local recurrence (months)			0.6973
<24	20	57.4 (35.0–77.2)	
≥24	9	60.0 (25.4–86.9)	
Site of local recurrence			0.8180
Bone	11	53.0 (25.0–79.2)	
Soft tissue	18	62.5 (37.7–82.1)	
Surgery for local recurrence			0.2070
Curettage	3	33.3 (4.3–84.6)	
Resection or amputation	26	61.8 (41.2–78.8)	
Denosumab administration before surgery for local recurrence			0.9573
No	27	59.3 (39.4–76.6)	
Yes	2	50.0 (5.9–94.1)	
Distant metastases			0.1548
No	19	69.7 (44.5–86.8)	
Yes	10	37.5 (13.5–69.7)	
Malignant transformation			0.2416
No	27	63.5 (43.4–79.9)	
Yes	2	0	

GCTB, giant cell tumor of bone. CI, Confidence interval.

## Data Availability

The datasets generated, analyzed, or both during the present study are not publicly available because of privacy issues but are available from the corresponding author upon reasonable request.
